# In Silico Dosimetry Study of Tc99m-Tetrofosmin in Children Using a Novel PBPK Model in Humans Built from SPECT Imaging Data

**DOI:** 10.1007/s11095-022-03412-w

**Published:** 2022-10-19

**Authors:** Christos Kaikousidis, Aristides Dokoumetzidis

**Affiliations:** 1 Department of Pharmacy, National and Kapodistrian University of Athens, Panepistimiopolis, 15771 Athens, Greece; 2 Pharma-Informatics Unit, Athena Research Center, Athens, Greece

**Keywords:** dosimetry, extrapolation, PBPK, radiopharmaceutical

## Abstract

**Purpose:**

The aim of this work is to develop a Physiologically Based Pharmacokinetic model (PBPK) for the radiopharmaceutical Tc99m-Tetrofosmin in humans, from literature SPECT imaging data, to carry out in-silico dosimetry studies in children and extrapolate dosing.

**Methods:**

A whole body PBPK model was developed from literature data from humans of Tc99m-Tetrofosmin tissue distribution. A data driven approach to estimate partition coeffects, permeability parameters and clearances was carried out, while some parameters were determined using a standard in silico PBPK method. Paediatric PK data for all tissues were simulated by changing the physiological parameters from the adult to paediatric values. Absorbed and effective doses for children of all ages were calculated using S-values from literature of Tc99m that have been computed from anthropomorphic phantoms.

**Results:**

Using the results from each tissue, satisfactory goodness-of-fit was achieved, assessed by visual inspection and a coefficient of determination of R^2^ = 0.965 while all estimated parameters had good standard errors. Paediatric simulations of Tetrofosmin distribution showed that paediatric profiles are not very different to the those of adults. The effective doses per unit of administered activity for 15 yo, 10 yo, 5 yo and 1 yo children were calculated to be 1.2, 1.7, 2.6 and 4.8 times higher, respectively than the adult value. Based on these calculations maximum administered activity scale more than proportionately to body weight.

**Conclusions:**

A PBPK model of tetrofosmin in adults has been developed from SPECT imaging data and was extrapolated to conduct in-silico dosimetry studies in children of all ages.

**Supplementary Information:**

The online version contains supplementary material available at 10.1007/s11095-022-03412-w.

## Introduction

It has been widely reported that children are not small adults. Both the relative sizes of their organs as well as developmental factors, alter the pharmacokinetics and limit the use of a simple “mg-per-kg” approach for drug dosing. For radiopharmaceutical diagnostics used in applications such as SPECT imaging, it is important to recommend doses that are well below a safety limit determined by the radioactivity that a patient is exposed to. This is done by carrying out dosimetry studies that quantify radioactivity in each organ with respect to time, by doing whole body scans in volunteers. From these images the kinetics of the compound are calculated and the radioactivity exposure in each tissue is determined, which consists of what is contributed by the presence of the drug in that tissue and what is radiated to that tissue by all other tissues. Finally, the total health hazard, called “effective dose”, is calculated by averaging up the radioactivity exposure in all tissues. These steps are carried out with use of complex anthropomorphic phantoms [1], that are realistic computational models of the human anatomy.

Phase 1 studies such as full body scans are difficult to conduct on healthy volunteers, as this is considered unethical, while if this is conducted in patients in a phase 2 rationale it is feasible but still difficult [2]. Therefore, dosage recommendations for radiopharmaceuticals in children largely use kinetics of adults. Anthropomorphic phantoms for children of all ages exist to carry out the calculations of the “effective dose”.

One way to address this problem is by modelling and simulation (M&S) which has offered in the recent years ways to carry out virtual or in silico clinical trials with various degrees of impact depending on the application. One of the important tools or M&S methodologies in drug development is Physiologically Based Pharmacokinetic (PBPK) models [3], which are mathematical models parametrized appropriately, using mechanistic information that allow extrapolations and are anatomically and physiologically constrained. From a methodological point of view PBPK models are developed using either *in vitro* or preclinical data and then extrapolated to humans, since data from humans that offer mechanistic information (e.g. drug levels from individual tissues) are difficult or impossible to obtain.

Effort has been made to bridge the gap between PopPK and physiological models, such that the latter can be informed by clinical and not only by *in vitro* or preclinical data. Utilization of clinical data to inform PBPK models and incorporation of variability is useful since in this way physiological parameters can be associated to the source of observed variability, and more accurate predictions and extrapolations can be made to special patient conditions or patient populations that can be difficult or indeed impossible to gain data from, such as paediatric or geriatric patients, or various scenarios of co-medications and comorbidities.

One way to do this is to develop PBPK models directly in humans by using information from tissues. Invasive or non-invasive techniques that can sample drug concentration from tissues and fluids exist such as biopsies, Fine Needle Aspiration and microdialysis. However, these are invasive techniques that can be part of a therapeutic intervention or diagnostic procedure while pharmacokinetics is a secondary endpoint. Also, they offer drug concentrations in specific tissues or fluids of particular interest, not PK in all tissues as needed for Whole Body PBPK models. A technique that can do this is radiolabelling with imaging. This includes Positron Emission Tomography for some limited applications and scintigraphy.

The aim of this work is to develop a PBPK for the radiopharmaceutical Tc99m-Tetrofosmin in humans, from literature SPECT imaging data, in order to extrapolate dosing to children and essentially conduct an in silico dosimetry study in children.

## Methods

### Datasets

Literature data from healthy male volunteers [4] of Tc99m-Tetrofosmin tissue distribution after IV administration of a dose (3.7-4.7 mCi, mean 4.1 ± 0.2 mCi), were available for blood, urine and compact organs, i.e. heart, lung, liver, gallbladder, kidney, thyroid and GI. The data, represent the average percent of activity from 12 volunteers (from 2 clinical centres) while individual data were not available. Also, the data were corrected for background and decay taking into account the half-life of Tc99m. The organs had been measured by pixel counting from imaging data and included 7 time points up to 24 h, namely at 5, 30 min, 1, 2, 4, 8 and 24 h post-injection (an additional time at 48 h was available for urine) and all patients had the same time points. These corresponded to the beginning of the scan, a procedure that was reported to last 18 minutes in one of the two centres and 20-26 min (avg 23 mins) in the other centre averaging to 20.5 mins. Since this is not a negligible duration it would be wrong to assume that the signal measured at each time point, represent the value at the start or end of the measuring process. Therefore, the % activity measured is considered to be the value at the middle of this duration (~10 min) and was introduced as a time lag to the available data points.

Distribution in the brain is negligible as the drug is known not to cross the BBB. All measurements were available as a percentage of the administered dose and are tabulated in Tables S1 – S3 in the Supplementary material.

### Model Structure

**Fig. 1:**
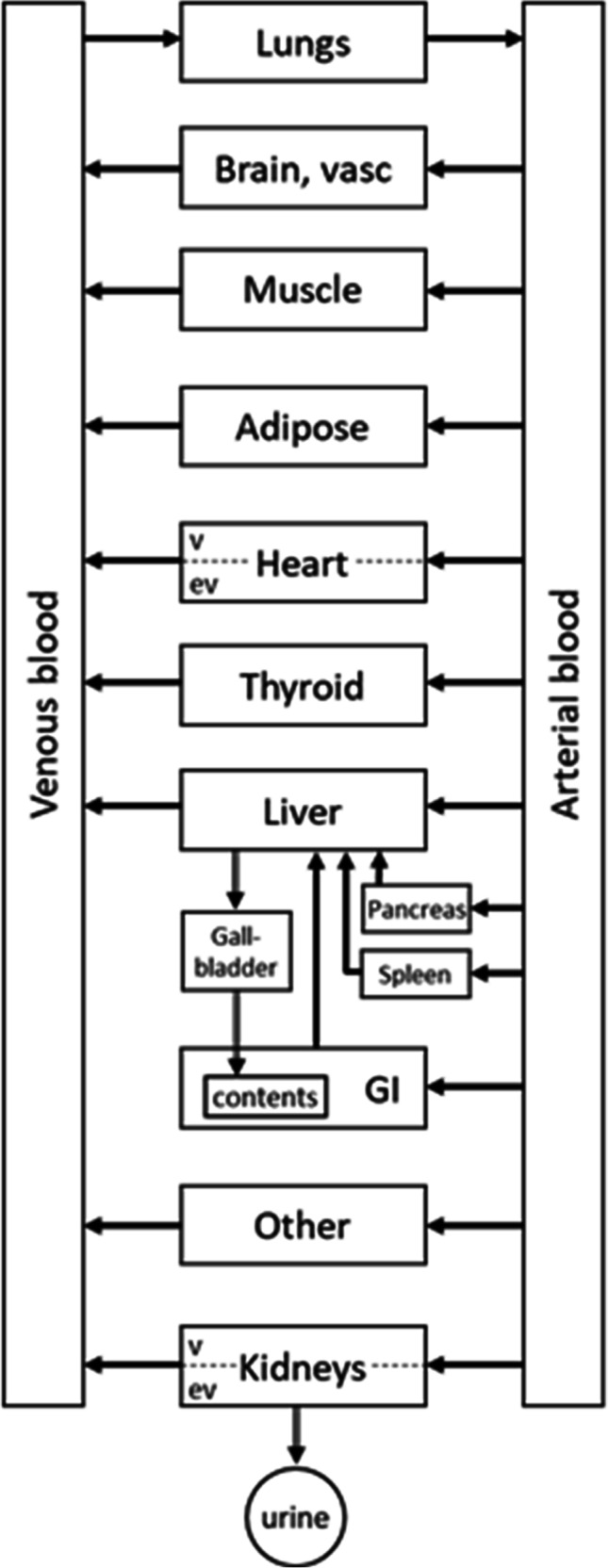
Schematic diagram of the tetrofosmin PBPK model structure.

A PBPK model was considered with explicit compartments for the following tissues: Arterial and venous blood, heart, lung, liver, gallbladder, kidneys, GI, thyroid, skeletal muscle and adipose, as pictured in Fig. 1. Furthermore a vascular and extravascular compartment was considered for each of tissue, namely for heart, lung, liver and kidneys while the model selection between this type of model and the simpler model with a single compartment was carried out by goodness of fit criteria. Model parameters that were estimated included the partition coefficients, permeability parameters as well as clearances, while physiological parameters such as blood flows and organ volumes were taken from literature [5], Tables S4-S5 in the Supplementary materials. The Kp parameters for muscles, adipose, spleen, pancreas and GI were calculated using standard PBPK in silico methods namely the Rogers and Rowland method, since no usable data for these tissues were available. The inputs for the calculations appear in the Table I, while the equations can be found in [6].

**Table I:** Input Parameters for Rogers and Rowland equations found in [6] to Calculate the Kp Values for Muscle, Adipose, GI, Spleen and Pancreas

Attribute	value
Molecular Weight(g/mol)	898.86
LogPo:w	1.14
Compound Type	Monoprotic base
pKa	8.3
Blood/Plasma ratio	0.8
Fu (fraction unbound)	0.7353
Distribution model	Rogers and Rowland [6]

Due to the nature of the data, being percent of radioactivity administered coming from a specific region and not actual tissue concentration, as in classic PBPK models, the following assumptions were made: (a) The amount of drug in the GI is considered to include the GI contents and in fact it is dominated by it, since the drug is accumulated in the GI by enterohepatic recirculation through the bile [13]. (b)The blood in the heart chambers is considered to contribute to the measured radioactivity found in the data.

### Organ Specific Models

The differential equations for the PBPK model follow, describing the fraction of administered dose at time t in each organ or tissue, A_organ_(t). The various quantities and parameters values that appear in the equations are the following: Q_organ_ is the blood flow in that organ, V_organ_ is the volume of the organ, K_p,organ_ is the partition coefficient in that organ, i.e. the ratio of the concentration in the organ over the blood concentration at steady state and R is the blood to plasma ratio of the drug taking the fixed value 0.8, which was calculated using Simcyp PBPK software (Simcyp, Certara).

*Arterial blood*

$$\frac{d{A}_{art}}{dt}={Q}_{lung}\left(\frac{A_{lung}}{K_{p. lung}{V}_{lung}}-\frac{A_{art}}{V_{art}}\right)$$

*Venous blood*

$$\frac{d{A}_{ven}}{dt}= input-{Q}_{lung}\frac{A_{ven}}{V_{ven}}+{Q}_{muscle}\frac{R\times {A}_{muscle}}{K_{p. muscle}{V}_{muscle}}+{Q}_{heart}\frac{A_{heart,v}}{V_{heart.v}}+{Q}_{brain}\frac{A_{brain,v}}{V_{brain.v}}+{Q}_{liver}\frac{R\times {A}_{liver}}{K_{p. liver}{V}_{liver}}+{Q}_{thyroid}\frac{R\times {A}_{thyroid}}{K_{p. thyroid}{V}_{thyroid}}+{Q}_{adipose}\frac{R\times {A}_{adipose}}{K_{p. adipose}{V}_{adipose}}+{Q}_{kidney}\frac{A_{kidney,v}}{V_{kidney,v}}+{Q}_{other}\frac{R\times {A}_{rest}}{K_{p. other}{V}_{other}}$$

*Lungs* are considered to follow a perfusion limited model:

$$\frac{d{A}_{lung}}{dt}={Q}_{lung}\left(\frac{A_{ven}}{V_{ven}}-\frac{A_{lung}}{K_{p. lung}{V}_{lung}}\right)$$

*Heart* is considered to follow a permeability limited model and is divided into vascular (v) and extravascular (ev) compartments

$${\displaystyle \begin{array}{c}\frac{d{A}_{heart.v}}{dt}={Q}_{heart}\left(\frac{A_{art}}{V_{art}}-\frac{A_{heart.v}}{V_{heart.v}}\ \right)+{PS}_{heart}\left(\frac{R\,\times\, {A}_{heart. ev}}{K_{p. heart}{V}_{heart. ev}}-\frac{A_{heart.v}}{V_{heart.v}}\right)\\ {}\frac{d{A}_{heart. ev}}{dt}=-{PS}_{heart}\left(\frac{R\,\times\, {A}_{heart. ev}}{K_{p. heart}{V}_{heart. ev}}-\frac{A_{heart.v}}{V_{heart.v}}\right)\end{array}}$$

Where PS_heart_ is a permeability x surface parameter. The heart total amount is the amount in the vascular and extravascular spaces plus the blood in the heart chambers

$${A}_{heart, total}={A}_{heart,v}+{A}_{heart, ev}+{V}_{heart\ chambers}\left(\frac{A_{ven}}{V_{ven}}+\frac{A_{art}}{V_{art}}\right)$$

For muscles (perfusion limited), A_muscles_

$$\frac{d{A}_{muscle}}{dt}={Q}_{muscle}\left(\frac{A_{art}}{V_{art}}-\frac{R\times {A}_{muscle}}{K_{p. muscle}{V}_{muscle}}\right)$$

For brain, A_brain,v_, follows the same model as the heart and it is considered that PS_brain_ = 0 and therefore A_brain,ev_ = 0 always, since tetrofosmin does not cross the BBB.

The *hepatic* system comprises of the *Liver, Gallbladder, GI, Pancreas, Spleen* and the *GI contents* compartments

$${\displaystyle \begin{array}{c}\frac{d{A}_{liver}}{dt}=-\frac{Q_{liver}}{K_{p. liver}}\frac{R\,\times\, {A}_{liver}}{V_{liver}}+{Q}_{hepatic}\frac{A_{art}}{V_{art}}-{CL}_{liver}\frac{A_{liver}}{V_{liver}}+\frac{Q_{GI}}{K_{p. GI}}\frac{R\,\times\, {A}_{GI}}{V_{GI}}\ \\ {}+{Q}_{pancreas}\frac{R\,\times\, {A}_{pancreas}}{K_{p. pancreas}{V}_{pancreas}}+{Q}_{spleen}\frac{R\,\times\, {A}_{spleen}}{K_{p. spleen}{V}_{spleen}}\\ {}\begin{array}{c}\frac{d{A}_{gb}}{dt}={CL}_{liver}\frac{A_{liver}}{V_{liver}}-\frac{K_{gb}{A}_{gb}}{V_{gb}}\\ {}\frac{d{A}_{GI}}{dt}={Q}_{GI}\left(\frac{A_{art}}{V_{art}}-\frac{R\,\times\, {A}_{GI}}{K_{p. GI}{V}_{GI}}\right)\\ {}\begin{array}{c}\frac{d{A}_{GI, contents}}{dt}=\frac{K_{gb}{A}_{gb}}{V_{gb}}\ \\ {}\begin{array}{c}\frac{d{A}_{spleen}}{dt}={Q}_{spleen}\left(\frac{A_{art}}{V_{art}}-\frac{R\,\times\, {A}_{spleen}}{K_{p. spleen}{V}_{spleen}}\right)\\ {}\frac{d{A}_{pancreas}}{dt}={Q}_{pancreas}\left(\frac{A_{art}}{V_{art}}-\frac{R\,\times\, {A}_{pancreas}}{K_{p. pancreas}{V}_{pancreas}}\right)\end{array}\end{array}\end{array}\end{array}}$$

The total amount in the GI is

$${A}_{GI, total}={A}_{GI}+{A}_{GI, contents}$$

*Kidneys* are divided into vascular and extravascular compartments, while *urine* is also considered to accumulate.

$${\displaystyle \begin{array}{c}\frac{d{A}_{kidney,v}}{dt}={Q}_{kidney}\frac{A_{art}}{V_{art}}-{CL}_{renal}\frac{A_{kidney,v}}{V_{kidney,v}}-{Q}_{kidney}\frac{A_{kidney,v}}{V_{kidney,v}}\\ {}+{PS}_{kidney}\left(\frac{A_{kidney, ev}\times R}{K_{P, kidney}{V}_{kideny, ev}}-\frac{A_{kidney,v}}{V_{kidney,v}}\right)\\ {}\begin{array}{c}\frac{d{A}_{kidney, ev}}{dt}=-{PS}_{kidney}\left(\frac{A_{kidney, ev}\times R}{K_{P, kidney}{V}_{kidney, ev}}-\frac{A_{kidney,v}}{V_{kidney,v}}\right)\\ {}\frac{d{A}_{urine}}{dt}={CL}_{renal}\frac{A_{kidney,v}}{V_{kidney,v}}\end{array}\end{array}}$$

*Adipose* follows perfusion limited kinetics

$$\frac{d{A}_{adipose}}{dt}={Q}_{adipose}\left(\frac{A_{art}}{V_{art}}-\frac{R\times {A}_{adipose}}{K_{p. adipose}{V}_{adipose}}\right)$$

For thyroid, A_thyroid_ follows the same model as adipose.

### Modelling Procedure

For the human data, first, a model was developed first for each of the tissues, in an open loop mode, using an empirical forcing function as blood input for each of the respective tissue ODEs. More specifically, the available blood data were assumed to be venous blood and were fitted to an empirical multi-exponential model to be used as a forcing function for an open loop model of the lungs. Then the arterial blood exiting the lungs was considered as an input function for open loop models of all the other organs. For each tissue kinetics, a perfusion limited *vs* a permeability limited model was selected based on goodness of fit, mainly by visual inspection. Special cases were the liver and the kidneys, as shown in organ specific model equations. The open loop step was performed so that the appropriate model for each tissue can be selected and a first estimate of the drug specific model parameters, i.e. partition coefficients, Kp, permeability x surface area parameters, PS and clearances could be obtained.

Finally, a whole body, closed loop model was constructed with the ODEs of all tissues as well as two ODEs for the blood (arterial and venular) in a large system of 19 ODEs in total. In this closed loop model an extra compartment was added (A_other_) accounting for the other tissues not included explicitly in the dataset, mainly to maintain mass balance. In total, 11 parameters were re-estimated by fitting the model to all tissue data simultaneously. Parameter estimation was carried out in the software NONMEM (version 7.4, Icon plc.), and proportional error model was assigned to each of the responses for the maximum likelihood estimation, in the form, Y=F*(1 + ERR(N)), where Y is the observation, F is the prediction and ERR(N) is the random variable for the error for the Nth compartment, therefore a separate error term was estimated for each tissue.

The next step was to scale the model to paediatric patients of 1, 5, 10 and 15 years of age by changing appropriately the physiological parameter values, i.e. the organ volumes and blood flows. (Tables S4-S5). These values were obtained from [5, 12].

Kp values were kept the same for the paediatric models. This is a common choice for extrapolation of PBPK models across species [3] An alternative choice is to keep the Kpu constant, but for tetrofosmin, protein binding in blood is considered limited so the two options are not very different. Furthermore, here extrapolation is considered from adult humans to children therefore differences are not anticipated to be very pronounced. PS values and clearance values were scaled allometrically according to the following formula

1 $${PS}_{child}={PS}_{adult}{\left(\frac{V_{organ, child,v}}{V_{organ, adult,v}}\right)}^a$$

where *a* is an allometric exponent taking the value 2/3 for PS and 3/4 for clearance based on geometrical arguments and is often used in PBPK models [3]. The rationale behind these choices is that surface scales with a power of 2/3 with respect to volume (or size in general) while permeability remains constant. For clearance, an allometric law has been observed in nature where metabolic rates scale proportionately to the 3/4 of body or organ size and is a common choice for PBPK models in the absence of contrary information [3], particularly in paediatrics.

From the paediatric PBPK models, profiles of the tetrofosmin can be simulated in all tissues. Also the activity (*a*_organ_) of Tc99m in an organ can be simulated by multiplying the tetrofosmin profile (A_organ_), for a dose of 1 MBq, by the exponential radioactive decay of Tc99m which has a radioactive half-life of 6.02 hours (=361.2 mins). i.e.

2 $${a}_{organ}(t)={A}_{organ}(t)\cdot{e}^{-0.0192t}$$

The model was implemented both in MATLAB (version R2019b, Mathworks) and in NONMEM and was solved numerically using functions ode15s and ADVAN8, in the two softwares, respectively. Exploratory simulations as well as simulations for the paediatric models were carried out in MATLAB, while parameter estimation, was carried out, as mentioned, in NONMEM using the FO method setting. Standard errors where calculated using the $COV subroutine of NONMEM.

### Dosimetry

**Fig. 2:**
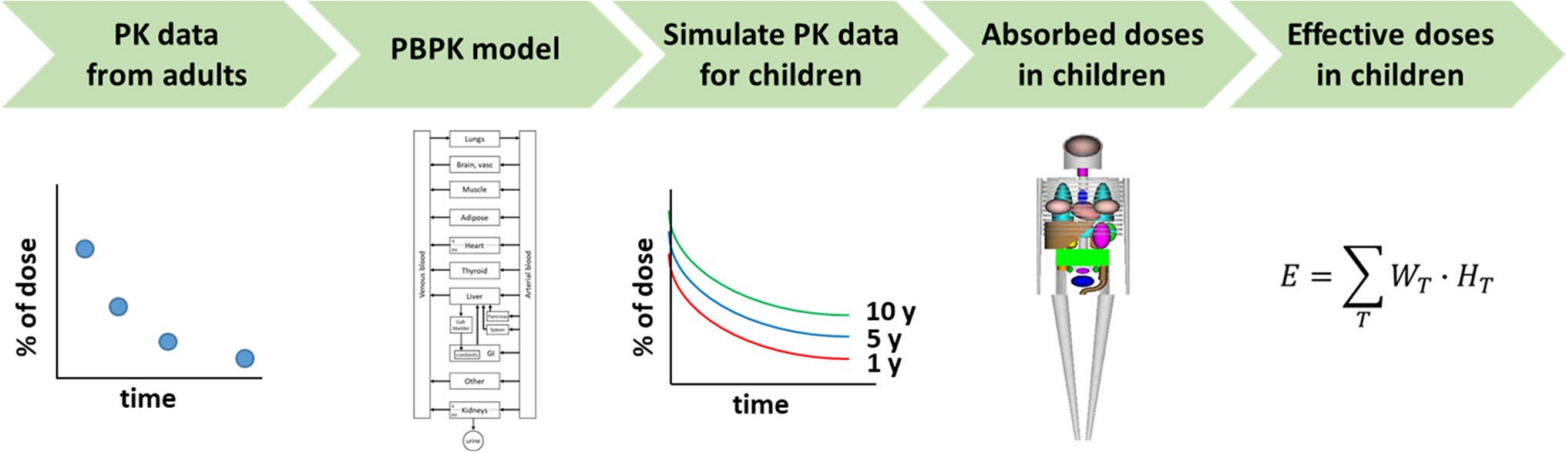
A schematic diagram of the workflow for the in silico dosimetry study in children. From the adult PK tetrofosmin data, a PBPK model for adults is constructed which is then scaled to produce paediatric PBPK models for children of different ages for tetrofosmin. With these PBPK models virtual PK data of tetrofosmin are simulated for children of all ages. Using appropriate S-values for Tc99m which have been produced from paediatric anthropomorphic phantoms, and combined to the PK data of tetrofosmin absorbed doses per activity unit, are generated for all organs for children of all ages. These absorpbed doses are averaged using appropriate weights, to effective doses per activity unit for children.

For all tissues, radiation dosimetry calculations were carried out to assess the safety of the proposed doses. This was performed following the MIRD procedure [7]. The workflow of entire procedure is summarised in Fig. 2. More specifically, the AUC values for the activity profiles, Eq. (2), as a fraction of the administered dose (N_S_, often referred to in literature as “residence times”), for all tissues were calculated and these were multiplied by Dose Fraction values, also called S values, according to the standard procedure recommended by MIRD. S values were obtained from the RADAR website [8] specifically for Tc99m and for ages 1, 5, 10, 15 years old and adult (Tables S6 to S10 in the Supplementary material). These parameters have been calculated from computational anthropomorphic phantoms of appropriate ages [9]. They calculate the radiation contribution to each organ coming from the organ itself as well as from all other organs, after the injection of a radioactive agent. Bladder emptying was considered to occur every 3 hours while the GI contents were considered to empty every 24 h.

The following formula was used to calculate the absorbed dose per activity unit administered (mGy/MBq) for all tissues

3 $${H}_T=\sum_S{N}_S\cdot S\left(T\leftarrow S\right)$$

Furthermore, the effective dose per administered activity can be calculated (E), in mSv/MBq units, by summing up the absorbed doses (H_T_) of all organs with appropriate weights (W_T_) according to ICRP103 2007 (Table S11 in Supplementary Material) [10].

4 $$E=\sum_T{W}_T\cdot {H}_T$$

### Dosing in Children

Maximum allowed children activities that can be administered were chosen based on safety, based on the assumption that the effective dose (mSv) in children should not exceed the equivalent effective dose produced by the maximum allowed administered activity for adults, reported in the Myoview SPC, being 1200 MBq [11]. That is, the effective dose after administration of 1200 MBq is calculated in adults and then the administered activity is determined in children that produces the same effective dose.

## Results

### PBPK Model Development for Adults

For the first step of the model development, where a separate model for each tissue is developed, a bi-exponential function was found to describe best the blood data and was used as a forcing function for the tissue open loop models, namely, *C*_*ven*_ = 25.98 *e*^−0.3004*t*^ + 0.3530  *e*^−3.533*t*^, where C_ven_ = A_ven_/V_ven_ .

Perfusion limited open loop models were used for most tissues as detailed in the ODEs found in the methods section, while permeability limited models including vascular and extravascular compartments were considered for the heart and the kidney. Gallbladder data were not used as they were very erratic. In the second step of the modelling procedure the resulting parameters were used as initial estimates for the final closed loop whole body PBPK model which made no use of the bi-exponential function and fitted all tissues and the blood simultaneously. This was found to sufficiently describe the data, including the blood. In order to measure the goodness of fit, the results from all tissues were used and the coefficient of determination (R^2^) for all datapoints was calculated giving R^2^ = 0.965 indicating a good fit. Also, to verify the goodness of fit graphically, a Predicted vs Observed plot was generated (Fig. S1, Supplementary material) which showed the predicted values are in accordance with the observed and no apparent pattern of point distribution was observed.

As mentioned, the Kp values for the muscle, adipose, GI, spleen and pancreas were calculated using the Rogers and Rowland equations, found in [6] and are shown in Table II using as input parameters the physicochemical properties of the drug shown in Table I.

**Table II:** Calculated Parameters using the Rogers and Rowland equations found in [6]

Parameter	Value
K_p,adipose_	1.04
K_p,GI_	0.26
K_p,spleen_	1.18
K_p,pancreas_	1.28

**Fig. 3:**
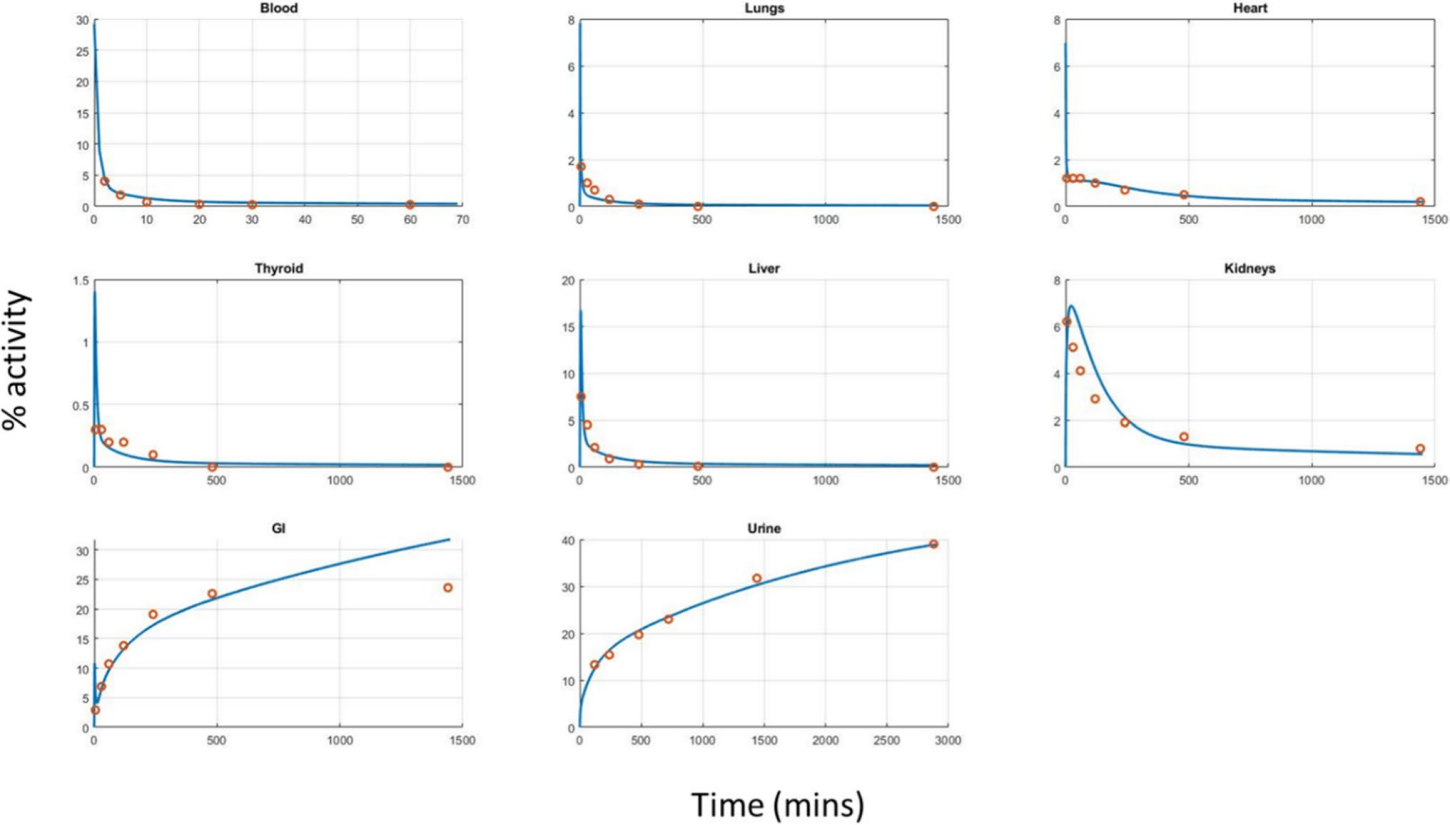
Profiles of the tetrofosmin model fitted to the data from adults for the various tissues.

The estimated model parameters are shown in Table III together with the corresponding standard errors which all take reasonably low values and the residual errors for each tissue are reported in Table IV. In Fig. 3 the simulated profiles of the model are plotted together with the data. Note that satisfactory fitting of the model to the data can be observed for all tissues.

**Table III:** Estimated Model Parameters with Standard Errors (SE) and Relative Standard Errors (RSE)

Parameter	Estimate	SE	%RSE
K_p,lung_	1.56	0.466	29.9
K_p,heart_	8.17	0.919	11.2
PS_,heart_ (L/min)	0.016	0.0021	13.1
K_p,kidney_	29.1	4.58	15.7
PS_,kidney_ (L/min)	0.247	0.09	36.4
K_p,liver_	1.88	0.53	28.2
K_p,thyroid_	13.4	3.89	29.0
K_p,other_	19.0	3.16	16.6
CL_renal_ (L/min)	0.156	0.009	5.8
CL_liver_ (L/min)	0.0683	0.0197	28.8
K_gb_ (min^−1^)	0.005	0.0029	58.0

**Table IV:** Proportional Residual Errors for each Tissue for Adult Model

Tissue	Value	%RSE
Venous Blood	0.409	29.09
Heart	0.128	29.29
Kidney	0.285	29.96
Liver	0.629	28.45
Lung	0.785	40.89
Thyroid	0.777	38.22
Gastrointestinal	0.222	27.70
Urine	0.045	29.77

### Simulations with the PBPK Model for Children

Using the developed model for adults paediatric models for ages of 1, 5 10, and 15 years, were constructed by changing the physiological parameters to match those for children (Tables S4-S5 in the Supplementary materials). Physiological parameters were taken from literature [5, 12] while drug related parameters were kept to the values estimated from adults. Note that permeability and clearance parameters were scaled rather kept constant according to the scaling relationships, Eq. 1, shown in Methods.

**Fig. 4:**
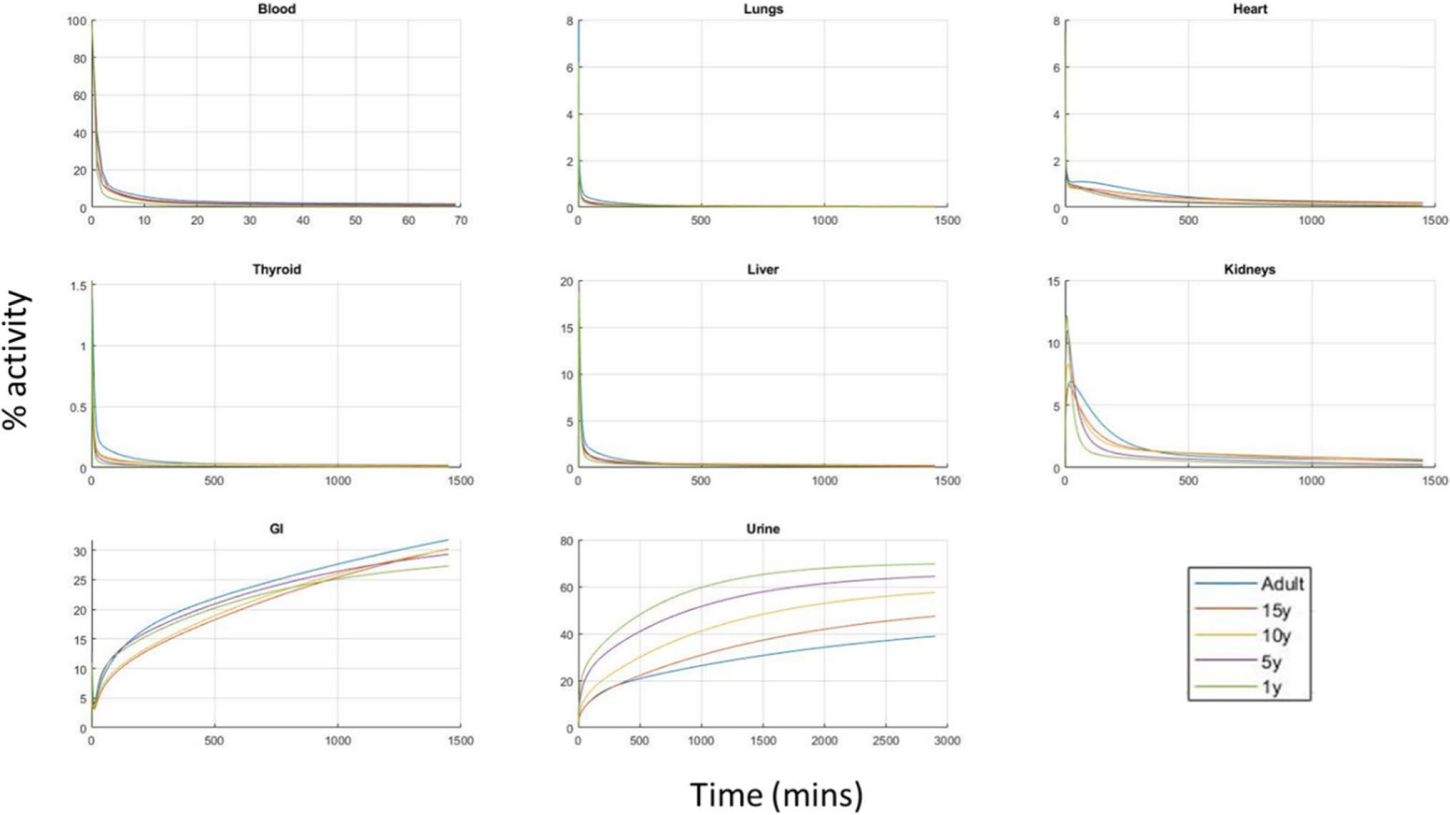
Profiles of the tetrofosmin model predictions for various tissues and various ages.

Using the paediatric models, simulations were carried out for the activity of Tc99m-Tetrofosmin in all tissues. In Fig. 4, comparative plots for tetrofosmin profiles are shown for ages 1, 5, 10, 15 years and adults.

### Dosimetry for Children

The PBPK models for children offer the kinetic models for activity that can calculate the Absorbed Doses in each organ, using Eq. 3 and the S-Values for Tc99m found in Tables S6-S10 in the Supplementary material section. These Absorbed Doses are calculated using anthropomorphic phantoms and account the radiation absorbed in each organ from the activity that originates from the organ itself, as well as from neighbouring organs. Table S12 (Supplementary) lists the Absorbed Doses for ages 1, 5, 10, 15 years and adults.

Furthermore, the health hazard caused from the exposure to the Absorbed Doses is summarised in the Effective Dose which averages the Absorbed Doses with appropriate weights (Table S11, Supplementary) according to Eq. 4, that reflect the susceptibility of the various organs to risks such as developing cancer [8]. The Effective Doses for ages 1, 5, 10, 15 years and adults are shown in the Table V together with the ratio of the Effective Dose for each age compared to adult’s. Note that for the 1yo the Effective Dose for the same administered dose is 4.8 fold, which means that a child of 1 yo will have the same health hazard with an adult when administered about 20% of the adult dose.

**Table V:** Effective Doses

Age	Effective Dose (mSv/MBq)	Ratio to adult
Adult	0.0059	1
15 y	0.0070	1.2
10 y	0.0102	1.7
5 y	0.0152	2.6
1 y	0.0285	4.8

Based on these calculations, the upper limit of the dose bracket can be calculated on safety grounds, namely from the Effective Dose that corresponds to the maximum allowed dose in adults as stated in the Myoview SPC which is 1200 MBq [11]. The doses that correspond to this effective dose (7.24 mSv) are reported in Table VI. Note that if these doses are not exactly proportional to body weight. Maximum doses that correspond to other ages, not reported in Table VI, can be calculated by linear interpolation.

**Table VI:** Recommended Activities for Children

Age	Max activity (MBq) based on effective dose	Max activity per body weight (MBq/kg)
Adult	1200	16.4
15 y	1011	18.1
10 y	694	21.7
5 y	466	24.5
1 y	248	24.8

## Discussion

Phase 1 studies in children are avoided and cannot be carried out in volunteers, therefore these are replaced by phase 2 studies that are carried out in paediatric patients but even these are difficult to conduct. Dosimetry studies of radiopharmaceuticals are such examples. On the other hand, in the recent years, the field of modelling and simulation has started to gain significant role in drug development with, lately, regulatory impact. The role of modelling in the past has been limited to describe in a more effective way existing clinical evidence, which evolved to the more impactful role of assisting deigning prospective studies. The future prospect of modelling in the form of in silico clinical trials is to waive or replace real clinical trials.

In this work an in silico dosimetry study in children has been carried out based on an adult dosimetry study. As outlined in Fig. 2, from the adult PK tetrofosmin data, a PBPK model for adults is constructed which is then scaled to produce paediatric PBPK models for children of different ages for tetrofosmin which are handled as if they were real generated clinical data to produce the effective doses per activity unit for children. The results of the study are reasonable and in many ways, expected.

One of the main clinical findings of this work is that the distribution of tetrofosmin between the different organs does not differ drastically for the different ages. This means that using the adult “residence times” in children for dosimetry is not a very bad approximation. This result is in line with dosimetry studies carried out directly in children which find satisfactory agreement with adult studies [2].

From a methodological point of view this study in one of the first to develop PBPK models directly in humans from imaging data. This offers significant advantages but also has limitations. The advantages include the fact that no extrapolation step is carried out from animals or from *in vitro* data. The model is developed directly in the species of interest, the human. An additional potential advantage, which has not been exploited here yet, is the prospect of including interindividual variability in the model, if the individual data were available. In the disadvantages we have to include that the fact this kind of imaging data carry large uncertainties. Also there were several missing tissues for which data were not available. This missing information needed to be replaced by assumptions, where the partition coefficients were calculated using in silico PBPK formulae, as used routinely in PBPK softwares such as Simcyp or PKSim. Some of these problems though are not disadvantages of the general rationale proposed, but are particular to the specific modelling exercise due to its retrospective character using old literature data. More modern studies may carry richer information, of better quality, with smaller experimental variabilities.

As mentioned above the ultimate ambition of in silico clinical trials, based on M&S approaches, is to be able to replace real clinical studies. This is what we call a regulatory application with a high impact. However, in order for a PBPK model to have a high regulatory impact it needs to be qualified for the intended use. This means that the predictive performance of the model needs to be demonstrated in a sufficient number of cases such that confidence is built that the model’s outcomes are reliable as described in EMA and FDA guidelines [14, 15]. This may include validation of the model with external datasets. One of the disadvantages of the present study is that no validation dataset has been used, for the extrapolation step since none was available. The PBPK model development step in adults may not need validation since it uses largely a data-driven approach as explained above.

## Conclusion

Overall, we have demonstrated a methodology to conduct an in silico dosimetry study in children from adult data by developing a PBPK model in adults which can be extrapolated to children of all ages, by changing the physiological parameters. Furthermore, this study is one of the first to develop a PBPK model from imaging data directly in humans. The results of the dosimetry study are reasonable and confirm findings in other products, i.e. Sestamibi [2] where the paediatric study was similar to that of adults. This approach is in line with the prospect of in silico clinical trials which we believe in the future will have a higher impact and may be in a position to replace real clinical studies, saving time and resources, while minimising risks for patients and volunteers.

## Supplementary Information

ESM 1 (DOCX 806 kb)

## Abbreviations

A_0_: Administered dose, MBq
A_organ_(t): Instantaneous fraction of administered dose distributed in *organ* at time t, %
A_organ,ev_(t): Instantaneous fraction of administered dose distributed in the extravascular compartment of *organ* at time t, %
A_organ,v_(t): Instantaneous fraction of administered dose distributed in the vascular compartment of *organ* at time t, %
CL_organ_: Clearance in organ, L/min
C_organ_(t): Instantaneous Fraction of administered dose distributed in *organ* per organ volume unit at time t, L^−1^
E: Effective Equivalent Dose in the whole body, mSv/MBq
H_T_: Absorbed Dose in the Target Organ, mGy/MBq
K_gb_: Flowrate of gallbladder contents emptying, L/min
K_p_,_organ_: tissue:blood partition coefficient for *organ*
N_s_: Residence time for organ S per unit of administered activity, sec
PS_organ_: Vascular to Extravascular Permeability- surface area product for *organ*, L/min
Q_organ_: *Organ* Blood Flow, L/min
R: Blood to plasma ratio
S(T ← S): Dose Fraction Value corresponding to the source (S) and target (T) organ, mGy/(MBq·sec)
V_organ_: *Organ* Volume, L
V_organ,ev_: *Organ* Extravascular Compartment Volume, L
V_organ,v_: *Organ* Vascular Compartment Volume, L
W_T_: Weight for converting the contribution of absorbed dose of organ T to the whole body effective dose
