# Supplementary material for: In Silico Dosimetry Study of Tc99m-Tetrofosmin in Children Using a Novel PBPK Model in Humans Built from SPECT Imaging Data
Source: Pharm Res. 2022 Oct 19;40(2):449–58. doi: 10.1007/s11095-022-03412-w (PMC9944701; doi:10.1007/s11095-022-03412-w)
Supplement: Supplementary file 1 — (DOCX 806 kb) [file 11095_2022_3412_MOESM1_ESM.docx]

**In silico dosimetry study of Tc99m-Tetrofosmin in children using a novel PBPK model in humans built from SPECT imaging data**

**Christos Kaikousidis and Aristides Dokoumetzidis**

**Department of Pharmacy, National and Kapodistrian University of Athens**

**and**

**Pharma-Informatics Unit, Athena Research Center**

**Correspondence**

**Aristides Dokoumetzdis:** [**adokoum@pharm.uoa.gr**](mailto:adokoum@pharm.uoa.gr)

**Supplementary Material**

**Input data and parameter values**

**Table S1.** Data from organs in humans [4]. All measurement are in % of administered dose.

| Time (min) | Heart | Lungs | Liver | Gallbladder | Kidneys | Thyroid | GI |
| --- | --- | --- | --- | --- | --- | --- | --- |
| 15 | 1.2 | 1.7 | 7.5 | 0.8 | 6.2 | 0.3 | 2.9 |
| 40 | 1.2 | 1 | 4.5 | 2.9 | 5.1 | 0.3 | 6.9 |
| 70 | 1.2 | 0.7 | 2.1 | 5.2 | 4.1 | 0.2 | 10.7 |
| 130 | 1 | 0.3 | 0.9 | 5.3 | 2.9 | 0.2 | 13.8 |
| 250 | 0.7 | 0.1 | 0.3 | 4.1 | 1.9 | 0.1 | 19.1 |
| 490 | 0.5 | 0 | 0.1 | 4.4 | 1.3 | 0 | 22.6 |
| 1450 | 0.2 | 0 | 0 | 1.5 | 0.8 | 0 | 23.6 |

**Table S2.** Data from blood in humans digitized and adapted from [4] to venular concentration.

| Time (min) | C_ven_ (% of dose/L) |
| --- | --- |
| 2 | 4.038 |
| 5 | 1.821 |
| 10 | 0.686 |
| 20 | 0.335 |
| 30 | 0.279 |
| 60 | 0.278 |

**Table S3**. Data from urine in humans digitized from [4]

| Time (min) | Urine (% of dose) |
| --- | --- |
| 120 | 13.3 |
| 240 | 15.4 |
| 480 | 19.7 |
| 720 | 23 |
| 1440 | 31.7 |
| 2880 | 39 |

**Table S4**. Organ blood flows [5, 12]

| Organ Blood Flows (L/min) | 1 y | 5y | 10y | 15y | Adult (Male) |
| --- | --- | --- | --- | --- | --- |
| Lung | 1.5 | 3.2 | 4.4 | 5.9 | 6.1 |
| Heart | 0.048 | 0.136 | 0.200 | 0.252 | 0.260 |
| Brain | 0.700 | 0.900 | 0.840 | 0.805 | 0.780 |
| Kidney | 0.230 | 0.577 | 0.854 | 1.335 | 1.335 |
| Liver | 0.306 | 0.867 | 1.140 | 1.365 | 1.660 |
| Liver (arterial) | 0.078 | 0.221 | 0.325 | 0.325 | 0.423 |
| GI Tract* | 0.180 | 0.510 | 0.750 | 0.840 | 0.975 |
| Spleen | 0.036 | 0.102 | 0.015 | 0.150 | 0.195 |
| Pancreas | 0.012 | 0.034 | 0.051 | 0.050 | 0.065 |
| Muscles | 0.072 | 0.212 | 0.429 | 0.941 | 1.110 |
| Adipose | 0.012 | 0.171 | 0.250 | 0.315 | 0.325 |
| Thyroid | 0.024 | 0.048 | 0.066 | 0.089 | 0.0915 |

*Stomach, Small Intestine, Large Intestine

**Table S5.** Organ volumes [5]

| Organ Volume (L) | 1y | 5y | 10y | 15y | Adult |
| --- | --- | --- | --- | --- | --- |
| Lung | 0.080 | 0.125 | 0.21 | 0.33 | 0.5 |
| Heart | 0.0981 | 0.219 | 0.549 | 0.659 | 0.807 |
| - Vascular | 0.0105 | 0.017 | 0.029 | 0.048 | 0.0693 |
| - Extravascular | 0.0395 | 0.067 | 0.110 | 0.181 | 0.2607 |
| - Heart Chambers | 0.0481 | 0.135 | 0.230 | 0.430 | 0.477 |
| Kidney Tissue | 0.070 | 0.11 | 0.180 | 0.250 | 0.31 |
| - Vascular | 0.0035 | 0.0055 | 0.0090 | 0.0125 | 0.0155 |
| - Extravascular | 0.0665 | 0.1045 | 0.1710 | 0.2375 | 0.2945 |
| Liver | 0.330 | 0.570 | 0.830 | 1.3 | 1.8 |
| Brain(Vascular) | 0.0475 | 0.0655 | 0.070 | 0.0710 | 0.0725 |
| Gallbladder | 0.002 | 0.017 | 0.030 | 0.053 | 0.068 |
| GI Tract Wall* | 0.155 | 0.392 | 0.665 | 0.93 | 1.260 |
| Spleen | 0.029 | 0.050 | 0.080 | 0.13 | 0.15 |
| Pancreas | 0.02 | 0.035 | 0.060 | 0.11 | 0.14 |
| Adipose | 3.8 | 5.556 | 8.6 | 12 | 18.2 |
| Muscles | 1.9 | 5.610 | 11 | 24 | 29 |
| Thyroid | 0.002 | 0.0034 | 0.008 | 0.012 | 0.020 |
| Blood | 0.500 | 1.4 | 2.4 | 4.5 | 5.1548 |
| - Arterial | 0.080 | 0.224 | 0.384 | 0.72 | 0.848 |
| - Venular | 0.322 | 0.903 | 1.548 | 2.9 | 3.4185 |
| Total Body Weight (kg) | 10 | 19 | 32 | 56 | 73 |

* Small Intestine, Large Intestine, Stomach

**Table S6.** S-values for Tc99m in adult (male), mSv/(MBq*sec) [8]


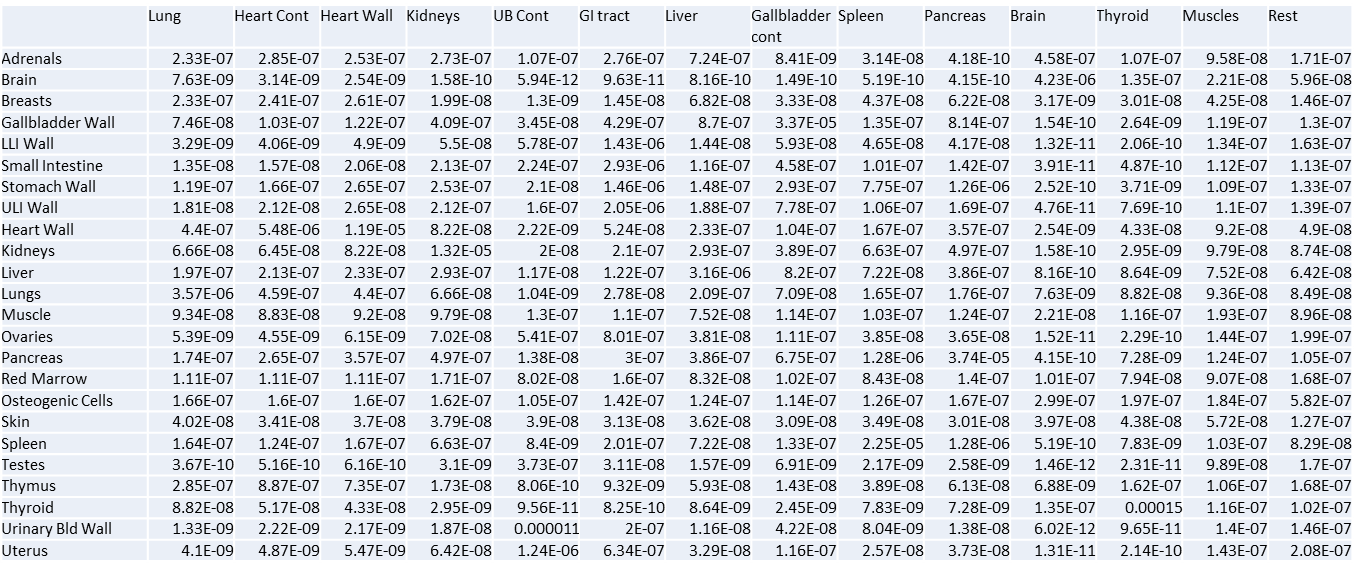


**Table S7**. S-values for Tc99m in 15 year old child (male), mSv/(MBq*sec) [8]


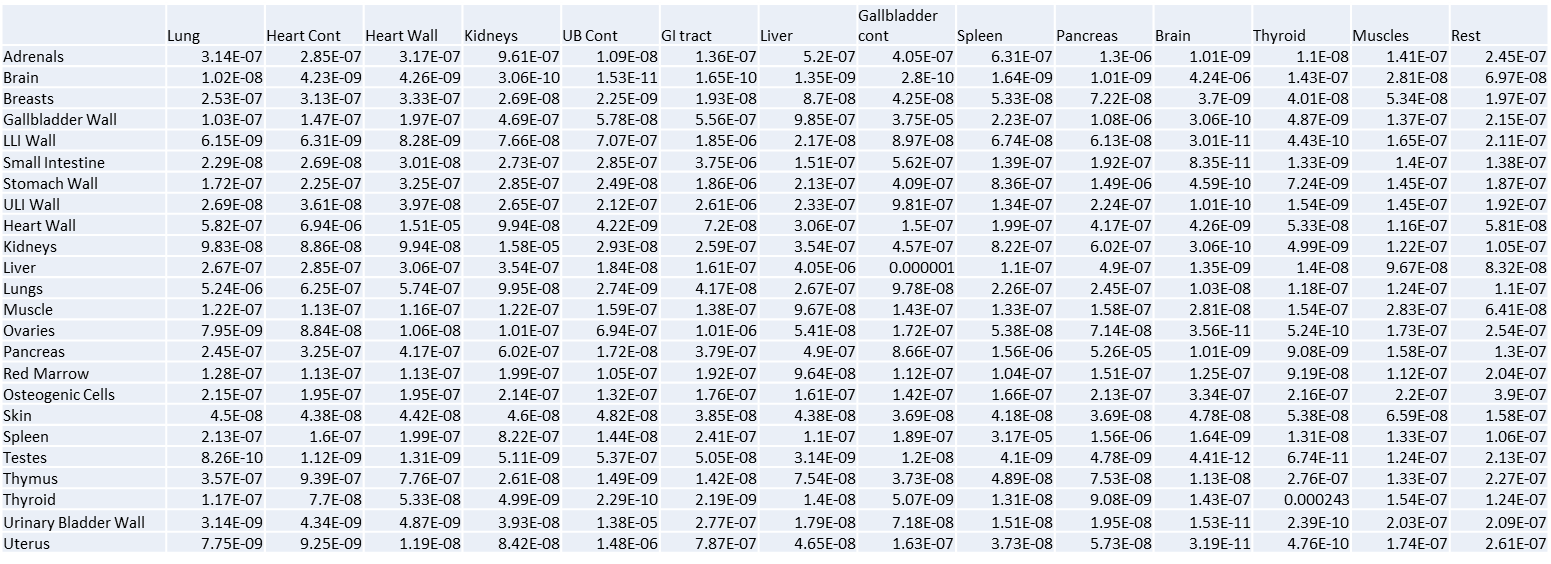


**Table S8.** S-values for Tc99m in 10 year old child, mSv/(MBq*sec) [8]


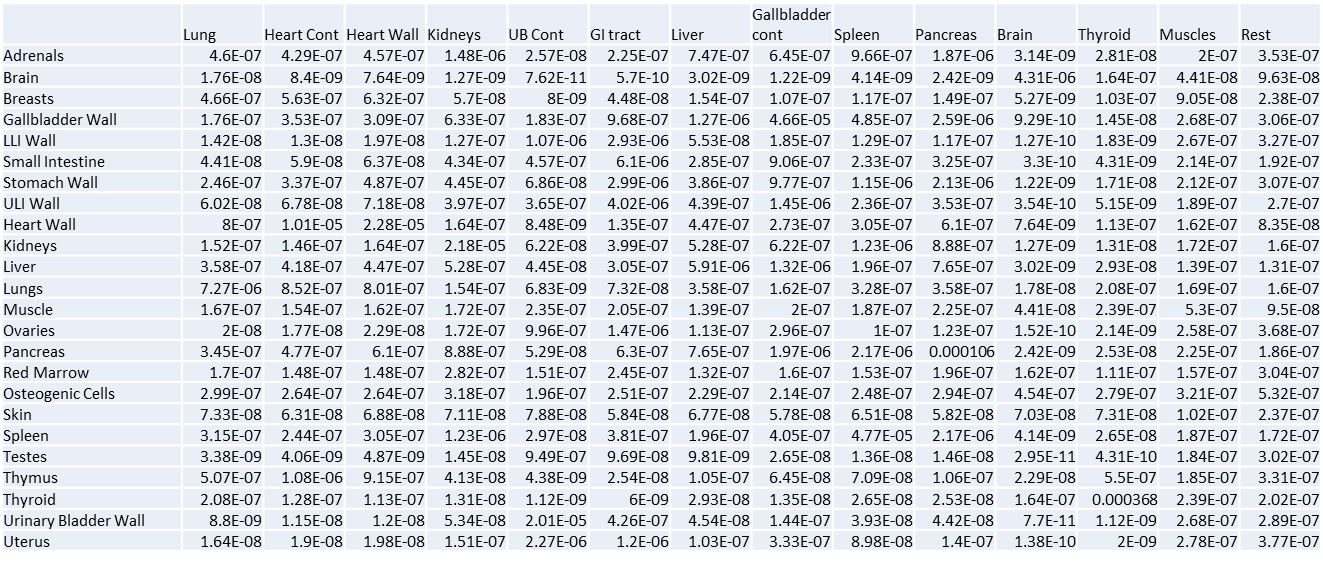


**Table S9.** S-values for Tc99m in 5 year old child, mSv/(MBq*sec) [8]


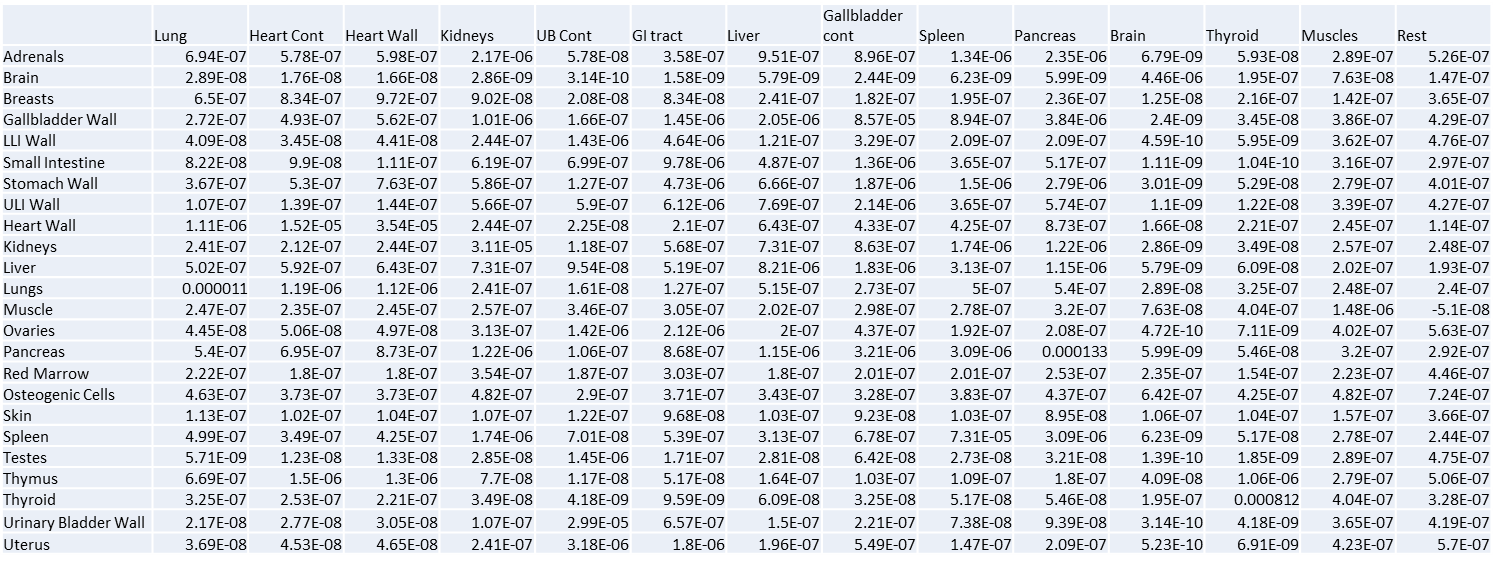


**Table S10.** S-values for Tc99m in 1 year old child, mSv/(MBq*sec) [8]


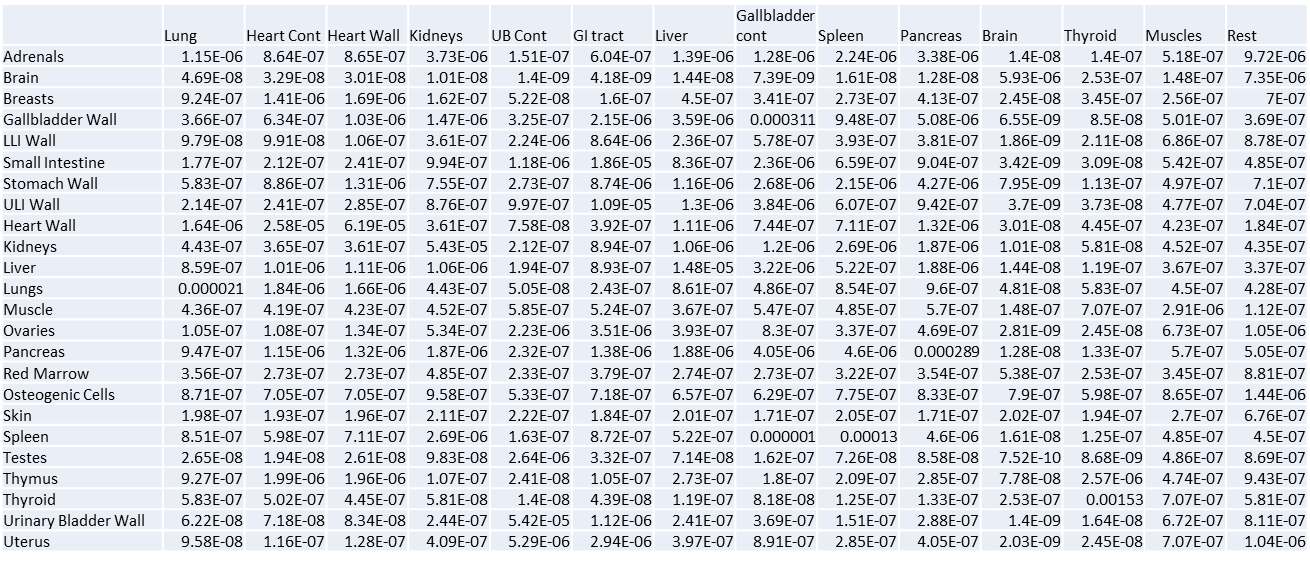


**Table S11.** Weights for the calculation of Effective Dose taken from [10] and adjusted to match the organs listed in the S-value tables (S6-S10)

| Organ | Weight |
| --- | --- |
| Adrenals | 0.02 |
| Brain | 0.01 |
| Breasts | 0.12 |
| Gallbladder Wall | 0.02 |
| LLI Wall | 0.06 |
| Small Intestine | 0.02 |
| Stomach Wall | 0.12 |
| ULI Wall | 0.06 |
| Heart Wall | 0.02 |
| Kidneys | 0.02 |
| Liver | 0.04 |
| Lungs | 0.12 |
| Muscle | 0.02 |
| Ovaries | 0 |
| Pancreas | 0 |
| Red Marrow | 0.12 |
| Osteogenic Cells | 0 |
| Skin | 0.01 |
| Spleen | 0 |
| Testes | 0.08 |
| Thymus | 0 |
| Thyroid | 0.04 |
| Urinary Bladder Wall | 0.04 |
| Uterus | 0 |

**Results**

**Table S12.** Organ Absorbed Doses in mGy/MBq

| Organ | 1 y | 5 y | 10 y | 15 y | adult |
| --- | --- | --- | --- | --- | --- |
| Adrenals | 0.10603823 | 0.009934 | 0.007814 | 0.005698 | 0.004935 |
| Brain | 0.07596248 | 0.001801 | 0.001478 | 0.001164 | 0.000975 |
| Breasts | 0.00873652 | 0.004992 | 0.003933 | 0.003335 | 0.002471 |
| Gallbladder Wall | 0.06327225 | 0.027893 | 0.017134 | 0.012147 | 0.010723 |
| LLI Wall | 0.05825359 | 0.033133 | 0.020793 | 0.013319 | 0.011328 |
| Small Intestine | 0.11027051 | 0.061051 | 0.03563 | 0.021988 | 0.019307 |
| Stomach Wall | 0.05751678 | 0.033109 | 0.021023 | 0.013141 | 0.011124 |
| ULI Wall | 0.06947442 | 0.041502 | 0.025855 | 0.017052 | 0.014624 |
| Heart Wall | 0.01140011 | 0.007328 | 0.005398 | 0.003992 | 0.003701 |
| Kidneys | 0.03183302 | 0.023196 | 0.018539 | 0.013521 | 0.012469 |
| Liver | 0.01263818 | 0.008077 | 0.005652 | 0.003798 | 0.003297 |
| Lungs | 0.00748051 | 0.004581 | 0.003574 | 0.002734 | 0.002168 |
| Muscle | 0.00682407 | 0.003751 | 0.003712 | 0.002722 | 0.002786 |
| Ovaries | 0.03158815 | 0.019609 | 0.013714 | 0.009737 | 0.008255 |
| Pancreas | 0.01787834 | 0.011646 | 0.008754 | 0.005915 | 0.005054 |
| Red Marrow | 0.01183341 | 0.007333 | 0.006137 | 0.0046 | 0.003909 |
| Osteogenic Cells | 0.02026356 | 0.011363 | 0.009763 | 0.007698 | 0.010191 |
| Skin | 0.00840444 | 0.004966 | 0.003921 | 0.002845 | 0.002288 |
| Spleen | 0.01291872 | 0.008415 | 0.006423 | 0.004404 | 0.003714 |
| Testes | 0.01162562 | 0.006993 | 0.005295 | 0.003945 | 0.003101 |
| Thymus | 0.0110656 | 0.006584 | 0.005347 | 0.004092 | 0.003081 |
| Thyroid | 0.01993622 | 0.012795 | 0.009458 | 0.006675 | 0.005987 |
| Urinary Bld. wall | 0.02296531 | 0.015473 | 0.010866 | 0.007879 | 0.00694 |
| Uterus | 0.02877676 | 0.018271 | 0.012728 | 0.008848 | 0.007611 |


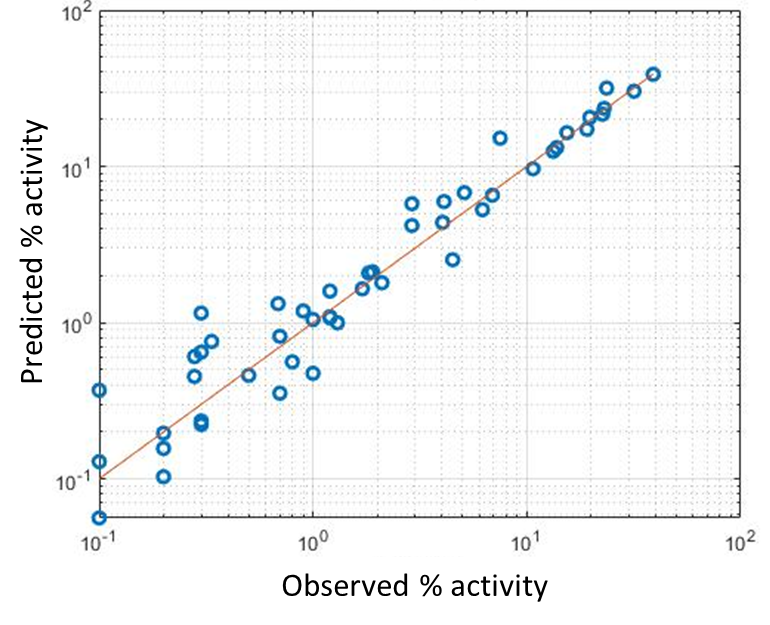


**Figure S1.** Predicted versus Observed % activity plot using datapoints from all tissues for the adult PBPK model. The red line is the identity line.
